# High Throughput Sequencing for the Detection and Characterization of RNA Viruses

**DOI:** 10.3389/fmicb.2021.621719

**Published:** 2021-02-22

**Authors:** Amy H. Fitzpatrick, Agnieszka Rupnik, Helen O'Shea, Fiona Crispie, Sinéad Keaveney, Paul Cotter

**Affiliations:** ^1^Food Biosciences, Teagasc Food Research Centre, Fermoy, Ireland; ^2^Shellfish Microbiology, Marine Institute, Oranmore, Ireland; ^3^Biological Sciences, Munster Technological University, Cork, Ireland

**Keywords:** high throughput sequencing, RNA viruses, environmental virology, amplicon sequencing, capture based probe hybridization, viral enrichment, RNA depletion

## Abstract

This review aims to assess and recommend approaches for targeted and agnostic High Throughput Sequencing of RNA viruses in a variety of sample matrices. HTS also referred to as deep sequencing, next generation sequencing and third generation sequencing; has much to offer to the field of environmental virology as its increased sequencing depth circumvents issues with cloning environmental isolates for Sanger sequencing. That said however, it is important to consider the challenges and biases that method choice can impart to sequencing results. Here, methodology choices from RNA extraction, reverse transcription to library preparation are compared based on their impact on the detection or characterization of RNA viruses.

## 1. Introduction

Many RNA viruses are of a global health concern from a One Health perspective, which is the intersection of human, animal and environmental health. Environmental transmission of these viruses, whether it be through food, water or recreational activities poses a risk for humans, plants and animals. It is important to adopt One Health principles for the surveillance of RNA viruses as environmental samples can (a) indicate hot spots for viral recombination, (b) serve as an important source of virus transmission, and (c) sequencing these samples allows us to pre-empt new RNA viruses and their variants of potential clinical concern. Viral persistence in the environment increases the opportunity for inter and intra-viral family recombination and increases virus-host exposure, factors that all contribute to the emergence of new viruses; that have the potential to cause large scale outbreaks. Non-enveloped viruses demonstrate remarkable persistence in the environment. Trans-kingdom virus interactions are thought to aid viral persistence in environmental settings, though this has been difficult to investigate, due to a lack of suitable cell culture systems. Furthermore, RNA viruses have high mutation rates as, unlike their DNA counterparts, most do not have a proofreading polymerase, though there are notable exceptions (Smith and Denison, 2013). These mutations can result in non-functional changes but can also enable the virus to evade the host immune system, through changing epitope conformation. Yet emerging RNA viruses are difficult to detect due to (a) lack of cell culture systems and (b) dependence on targeted molecular approaches. Second generation sequencing provided incremental improvements in the monitoring of environmental transmission, persistence and recombination but the costs quickly became prohibitive. This was in part due to the need to isolate viruses using cell culture or clone environmental samples for increased sequencing

resolution. In addition, the quantity of input RNA/DNA required to obtain high quality sequences. High throughput sequencing methods (bridge amplification, single molecular real time sequencing, and nanopore-based sequencing) have been widely applied in clinical settings but have had limited success for viral surveillance and aside from Flaviviruses (Zika virus, West Nile virus). There have been important contributions regarding RNA virology from environmental HTS applications (Alberti et al., 2017; Wolf et al., 2020) though HTS investigation of environmental transmission of pathogenic RNA viruses is still in its infancy. Furthermore, the comparatively small size of RNA virus genomes to competing genomic RNA, severely impacts the depth of coverage achievable, due to sequencing saturation. To summarize, RNA viruses are difficult to sequence and characterize using HTS due to (a) their genetic diversity, (b) lack of conserved regions across the genome of viruses and (c) short genome lengths.

## 2. Approaches

### 2.1. Targeted HTS

**Figure 1:**
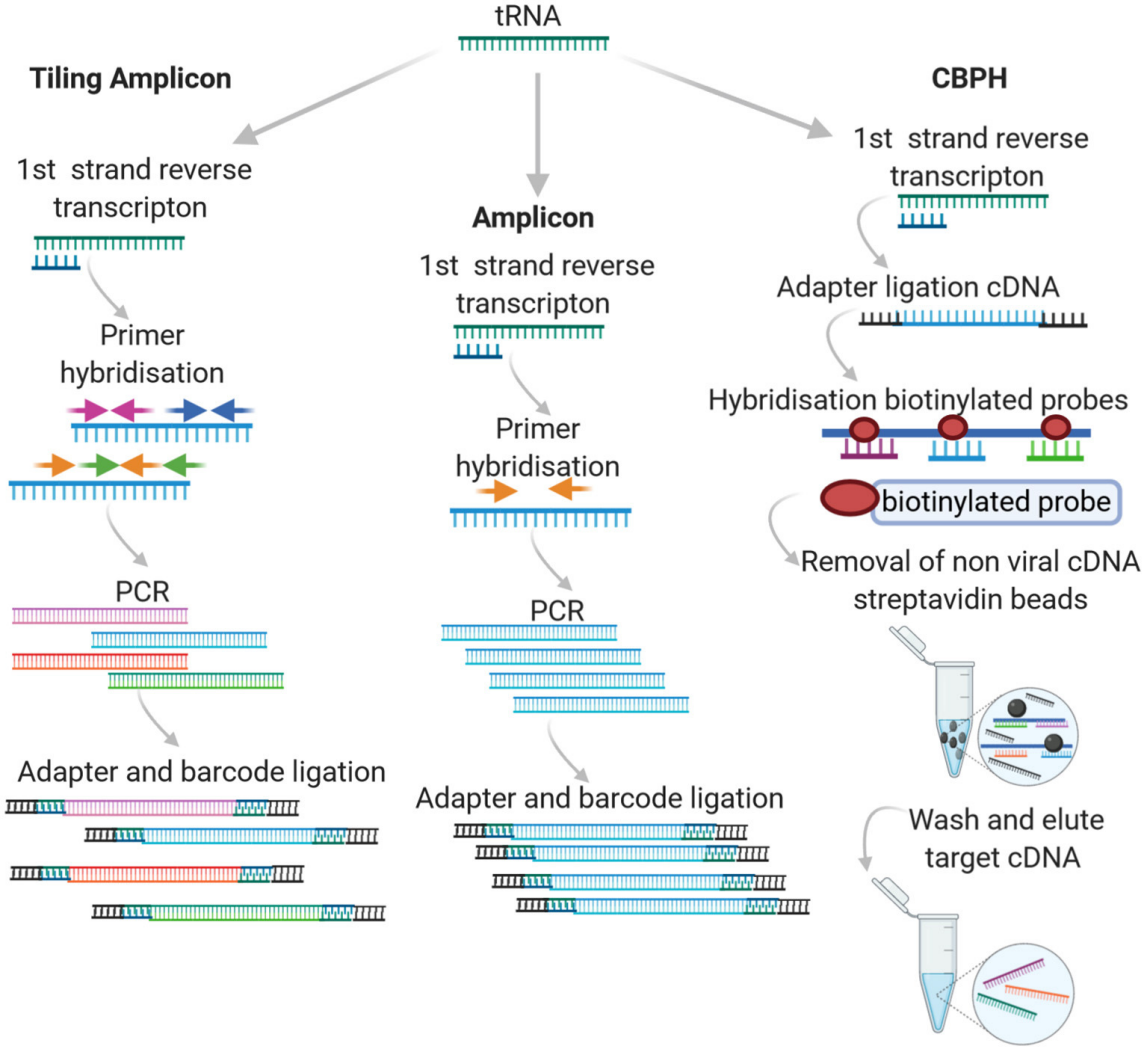
Targeted HTS approaches.

In High Throughput Sequencing (HTS), either a targeted or agnostic approach can be taken. Targeted sequencing infers that some level of knowledge is available with respect to the target in question and that the experimental design incorporates this prior knowledge, either through amplicon-based sequencing or probe capture hybridization. An overview of how the different approaches work can be seen in Figure 1.

#### 2.1.1. Amplicon Sequencing

One of the two most common approaches to targeted sequencing is amplicon sequencing. The approach involves amplification of a target genome fragment using specific primers before library preparation and sequencing. It is most often used for the study of diversity and structure of prokaryotic communities in a variety of hosts (human, animal and ecological niches). Often in this case, common PCR-based approaches target highly conserved rRNA genes, such as those encoding the 16S/18S and 28S subunits or the Internal transcribed spacer (ITS) between them. Unlike the 16S rRNA of bacteria, viruses lack universally conserved markers and genome plasticity. In particular, with respect to RNA viruses, this further contributes to the associated challenge, requiring a Family specific priming PCR approach. Amplicon based sequencing approaches, (particularly tiling or “jackhammer”) have been widely applied for the detection of RNA viruses with varying levels of success (Marston et al., 2013; Cruz et al., 2016; Cuevas et al., 2016; Hanke et al., 2016; Imamura et al., 2016b; Boonchan et al., 2017; Johnson et al., 2017; Parra et al., 2017; Quick et al., 2017; Hata et al., 2018; Lun et al., 2018; Suffredini et al., 2018; Wang et al., 2018; Cinek et al., 2019; Di et al., 2019; Fumian et al., 2019; Gradel et al., 2019; Eden et al., 2020; Fauver et al., 2020; Lu et al., 2020; Mancini et al., 2020). Tiling or “jackhammer” approaches involve designing a series of primers that generate short products across the whole target genome and can be Family or genus specific.

The success of amplicon sequencing with respect to RNA viruses is very dependent on the choice of primers. Like traditional Reverse Transcription Polymerase Chain Reaction (RT-PCR) amplification, primers can be designed to anneal to the most conserved sequences of the RNA virus genome(s) in question. In this case, a certain degree of validation is required. Confirmation of the PCR products *via* Sanger sequencing should be implemented for new primer sets to ensure specificity. There is a high likelihood that degenerate primer sets are required in order to account for virus divergence (Li et al., 2012). This highly targeted approach requires well-characterized viruses for which a number of viral genome sequences are available. In some cases, this may present a technical barrier, especially when developing amplicon sequencing methods to detect emerging infectious diseases, such as at the beginning of recent Ebola virus, Zika, and SARS-CoV-2 outbreaks when, initially, sequencing data was limited.

To circumvent this, Quick et al. (2017) developed a tiling amplicon algorithm called PrimalSeq to facilitate the design of primers that allow short amplicons to be generated across the target genome in a highly multiplexed assay. High quality (non-degenerate) sequences are required in order to design primers that target the entire length of the target genome. For this approach, the detection of recombinant viruses or intra-host nucleotide variants can be accurately detected by applying replicate sequencing, viral input greater than 1000 RNA virus copies and >400x genome coverage (Grubaugh et al., 2019). This approach has been widely applied to obtain whole genome sequences of emerging RNA viruses as it can work with samples with an expected high background host rRNA/mRNA, low concentrations of target viral RNA and limited diversity (Artic Network, 2021).

#### 2.1.2. Capture Based Probe Hybridization

Capture based probe hybridization (CBPH) requires prior knowledge of the specific sequence variants to be detected. Most capture-based methods use a tiling array approach, where 80 to 120-mer DNA or RNA probes are used to cover the length of the target genome/genomes. The probes typically have 10–50 bp regions between them, adopting a similar approach to the overlapping/jackhammer amplicon approach described above. Target enrichment is based on the biotinylated (or otherwise labeled) probe annealing to complementary sequences in the sample(s). The probes attach to previously fragmented genomic DNA/RNA and the targets are eluted, ligated, and prepared for the specific sequencing platform employed. Amplicon and capture probe hybridization tiling approaches have been widely applied as alternatives for whole genome sequencing of human genomic exons and viruses. In the latter case, this is in large part due to the challenge of sequencing small viral genomes in complex samples containing a high proportion of background host genomic DNA as well as, in some instances, bacteria/archaea.

CBPH was initially implemented to detect Single Nucleotide Variants (SNV) in human genomic exon studies. Various studies have applied this methodology to virus specific studies using widely available commercial kits with custom design options such as SureSelect XT Target Enrichment system, Illumina TruSeq RNA Access, or SeqCap Ez probe design with separate library preparation kit. More recently, the VirCapSeq-VERT and CATCH custom virus oligonucleotide panel have become available as a general tiling array for vertebrate viruses (summarized in Table 1). Metsky et al. (2019), Strubbia et al. (2019b), and Strubbia et al. (2020) are the only studies applying to CBPH to RNA viruses in non-clinical samples.

**Table 1:** Applied CBPH methods used for the characterization of viruses.

**Paper**	**Method/Kit**	**Virus family**	**Matrix**	**Detection limit**	**% reads mapped to virus**	**Depth of coverage**	**Genome coverage**	**Fold enrichment**
Depledge et al., 2019	SureSelect Target Enrichment +− Whole Genome Amplification	Herpesvirus	Saliva, blood, virus vesicles, cerebrospinal fluid, and tumor cell lines	250 ng–5 ug	52.84–99.48%	729–3,197	94–99%	
Duncavage et al., 2011	Custom probe panel and Illumina	Merkel cell polyomavirus (MCPyV)	FFPE tissue					40,000–107,000
Mate et al., 2015	TruSeq RNA Access kit	Ebola virus	Semen	NA			85.10%	100%
Briese et al., 2015	VirCapSeq-VERT + SeqCap RNA	Influenza A MERS-CoV, Enterovirus-D68, Dengue-3, WNV, Ebola virus, Cache Valley virus, Human herpesvirus 1	Blood and lung sample	100 gc/ ml	96.37–100%		13–5,230	100- to 10,000-fold increase
Wylie et al., 2015	ViroCap + SeqCap EZ	34 viral families, 337 species	Nasopharyngeal secretions, plasma, and stool		0.1–47.9%	0.01–19,097	0.8–100	Median fold increase 296–674
Miyazato et al., 2016	SeqCap EZ	HIV-1, human T-cell leukemia virus type-1	Cell culture supernatant		99.4–99.5%			657- to 13,418-fold enrichment
O'Flaherty et al., 2018	TruSeq RNA Access Library Prep kit Virus-specific probes	*Coronaviridae; Adenoviridae; Parvovirinae; Picornaviridae; Paramyxoviridae; Pneumoviridae; Orthomyxoviridae*	Virus dependent	Virus dependent	1.32–99.47%	0–102,724	1.8–100%	7,285-fold median increase in PTRs
O'Flaherty et al., 2018	TruSeq RNA Access Library Prep kit conserved viral group probes	*Coronavirinae, Adenoviridae, Pneumoviridae, Orthomyxoviridae*	Virus dependent	Virus dependent	0–99.22%			8,990-fold median increase in PTRs
Brown et al., 2016	SureSelect Target Enrichment	Caliciviridae, norovirus	Fecal	40 Ct	81%	12,227	100%	
van Beek et al., 2017	SureSelect Illumina	Caliciviridae, norovirus	Fecal		91%	4,679		
Thézé et al., 2018	SeqCap EZ	Zika virus	Serum	40 Ct		2,046–7,870	1.51–90.77%	
Metsky et al., 2019^*^	CATCH	356 species, 86 genera, 31 families	Plasma, serum, buccal swabs, urine, avian swabs, and mosquito pools	100 copies in 30 ng of background and 1,000 copies in 300 ng	84–95%	1.7–1,842		
Strubbia et al., 2019a^*^	SureSelect Target Enrichment	Caliciviridae, norovirus	Sewage and Fecal samples	NA		9.225–99.567		
Singanallur et al., 2019	SeqCap EZ	*FMDV*	Oral and nasal fluids, and rectal samples from pigs	>40.0 Ct	99.34%		93.70–96.25%	3,000-fold for FMDV detection
Strubbia et al., 2019b^*^	VirCapSeq-VERT	Caliciviridae, norovirus	Oysters, sewage					
Strubbia et al., 2020^*^	VirCapSeq-VERT	Caliciviridae, norovirus	Oysters	1000 gc/g DT				
Carbo et al., 2020	SeqCap EZ HyperCap (Roche, Basel, Switzerland)	Coronavirus, SARS-CoV-2, SARS-CoV, and MERS-CoV	Nasopharyngeal swabs	30 Ct		9041.4–46956.9	>91%	
Nasir et al., 2020	MyBaits Expert Virus SARS-CoV-2 panel (Arbor Biosciences, Ann Arbor, MI, USA)	*Coronavirus, SARS-CoV-2*	Mid-turbinate swabs	31.5 Ct		98.6–8214.4		

* *Environmental application*.

There is huge variability across CBPH assays applied for the genetic characterization of viruses, from oligonucleotide bait design approaches (RNA/DNA), matrices and target viruses. Studies attempting to capture a very wide viral diversity have used panels ranging from 300,000 to 2.1 million probes per assay (Duncavage et al., 2011; Wylie et al., 2015; O'Flaherty et al., 2018). There are cost and performance implications for utilizing these large panels, as the number of probes required dictates the cost of probe synthesis (Briese et al., 2015; Wylie et al., 2015; Metsky et al., 2019). These assays tend to use shorter oligonucleotides as each additional nucleotide increases the uniqueness of an oligonucleotide by a factor of four (Hendling and Barišić, 2019). This design difference results in varying genome coverage, as large generic panels have a greater propensity to capture viral diversity but fewer whole genomes, whilst more targeted assays result in improved genome coverage but less viral diversity. When designing or implementing a virus panel, the key point to consider is the research objective (Duncavage et al., 2011; Brown et al., 2016; Thézé et al., 2018) and the limited evidence available suggests that CBPH is a valuable tool for genotypic characterization of RNA viruses in non-clinical samples.

### 2.2. Agnostic Sequencing

In direct contrast to targeted sequencing, agnostic sequencing requires little prior knowledge of the target genome(s) though hopefully an understanding of the matrix and expected virome in question. For example, if the objective is to characterize human viruses of clinical concern in sewage, concentration and extraction methods must consider that sewage as a matrix will contain PCR inhibitors and that concentration methods may not concentrate both enveloped and non-enveloped RNA viruses of interest. Method validation and the inclusion of appropriate controls is necessary for interpretation of results and for setting quality control thresholds. As agnostic sequencing is not targeted, non-viral RNA can be captured during the library preparation and this can cause downstream issues. Indeed, obtaining sufficient genome coverage of virus RNA genomes against a background of host rRNA and mRNA is a challenge. Various approaches have been developed to enrich samples or to deplete rRNA as outlined below.

#### 2.2.1. Sequence Independent, Single Primer Amplification (SISPA)

Sequence Independent, Single Primer Amplification (SISPA) is a random priming method developed by Reyes and Kim (1991). SISPA involves directional ligation of oligonucleotide(s) to a target population of blunt ended DNA molecules. The common end sequence allows one strand of the double-stranded primer to be used in repeated rounds of annealing, extension and denaturation in the presence of a high-fidelity polymerase. SISPA has been used for the discovery of new viral agents, particularly in the veterinary field (Moser et al., 2016; Chrzastek et al., 2017; Myrmel et al., 2017; Cholleti et al., 2018; Zhao et al., 2018a).

To date, there have been three comparative studies in which SISPA has been compared with other metagenomic methods. Kugelman et al. (2017) compared DNA shotgun metagenomics, RNA template metagenomics using random hexamers and Klenow fragments, amplicon sequencing, SISPA, poly(A) tail enrichment using TruSeq RNA kit and Circular resequencing (CirSeq). Parras-Moltó et al. (2018) compared multiple displacement amplification (MDA) and SISPA to sequence DNA viruses, while Goya et al. (2018) compared the use of various SISPA and random hexamers protocols, with and without rRNA depletion. These comparative studies used clinical samples, cell culture supernatant or plasmid material to assess method efficiency.

Of the numerous approaches they used, Kugelman et al. (2017) determined that SISPA resulted in the highest error increase (9.0-fold) compared to CirSeq or Illumina TruSeq RNA Access kit and SISPA-generated sequences demonstrated an increased number of transition events. The accumulation of these errors could falsely indicate sub-clonal diversity or veil true diversity. Parras-Moltó et al. (2018) found that SISPA-generated viromes displayed uneven coverage profiles, with high coverage peaks in regions with low sequence complexity. Bias induced by random amplification methods had a minor impact, with random hexamers being preferable to SISPA for DNA virus metagenomics. Conversely, when Goya et al. (2018) compared the performance of SISPA with random hexamers, they found that the best performance was achieved with SISPA compared to samples subjected to rRNA depletion prepared with the Nextera XT DNA library kit. The coverage profiles were different for each method, with random hexamers providing a more uniform distribution across the genome, albeit lower coverage. Despite the difference between these three studies, it is apparent that, as currently employed, SISPA is not suitable for the identification of SNVs due to the high number of transition events and uneven coverage of the target genome for both DNA viruses and negative strand RNA viruses studied.

#### 2.2.2. Rolling Circle Amplification (RCA)

**Figure 2:**
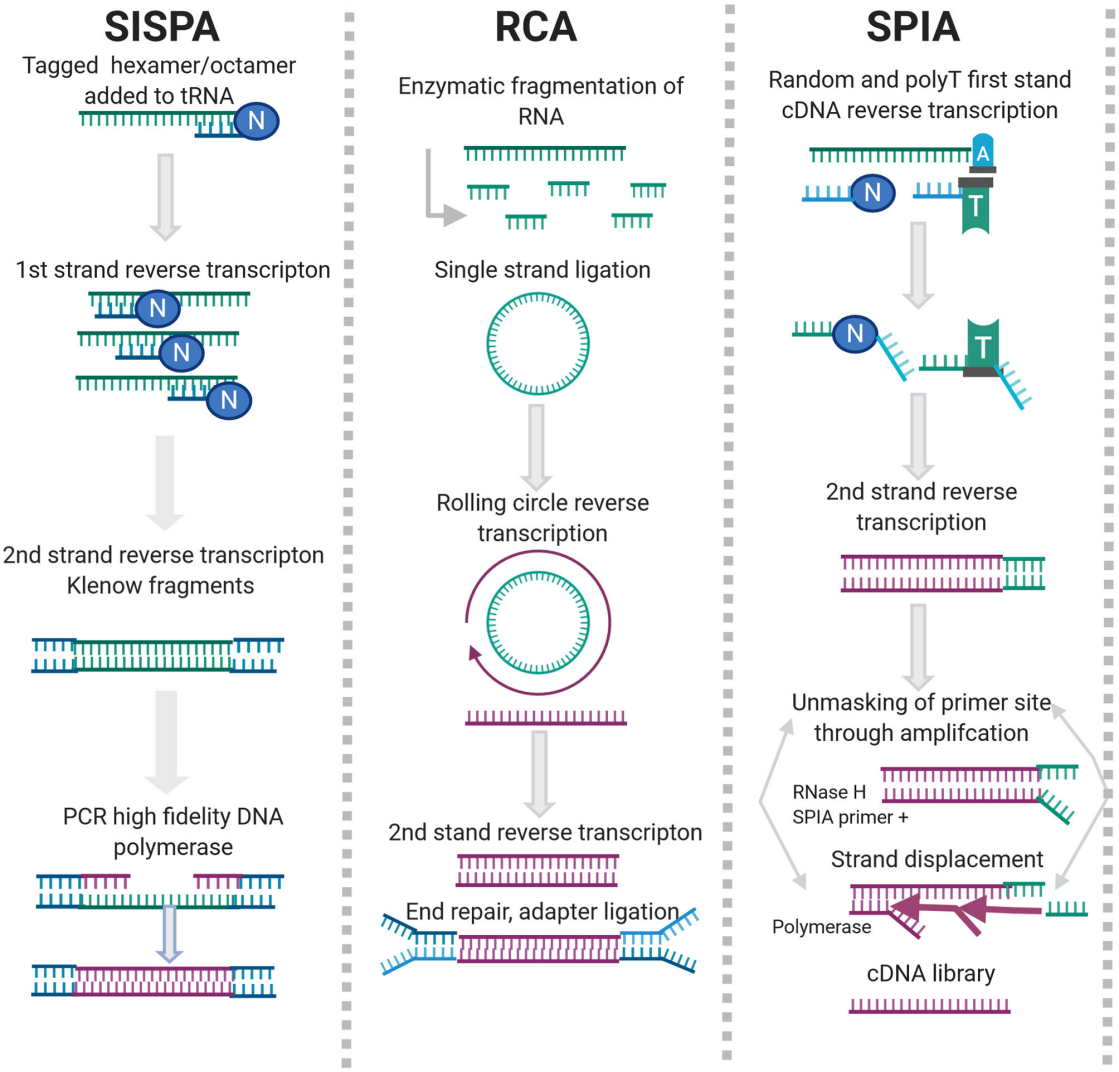
Enrichment methods for agnostic sequencing.

Rolling Circle Amplification (RCA) is an isothermal enzymatic process where a short DNA or RNA primer is amplified to form a long single stranded DNA or RNA using a circular DNA template and specific DNA or RNA polymerases, as can be seen in Figure 2.

So far only two studies have compared RCA-HTS methods or RCA to other shotgun metagenomics methods for use with DNA/RNA viruses or RNA templates (Kugelman et al., 2017; Sukal et al., 2019). Kugelman et al. (2017) found that CirSeq compared to other target enrichment methods (amplicon, SISPA), was the least error prone (Acevedo et al., 2014). However, Martel et al. (2013) found that the viral load required for CirSeq (1E + 3 IU/ml) is a limiting factor for the application of this method to clinical samples (Hepatitis B virus in serum). Sukal et al. (2019) used variations of RCA to detect and characterize integrated Badnavirus-like sequences in plant host species. Methods included; random-primed RCA primer spiked random-primed RCA, directed RCA and specific-primed RCA. Viral DNA amplified using the optimized directed RCA and specific-primed-RCA protocols showed an 85-fold increase in Badnavirus NGS reads compared with random-primed RCA, showing the benefit of target specific priming strategies.

#### 2.2.3. Ribosomal RNA

##### 2.2.3.1. Enrichment of Non-rRNA Transcripts

Ribosomal RNA (rRNA) is the most abundant species of RNA in most cells. For agnostic RNA virus sequencing of complex samples, its presence is problematic as a large number of non-viral reads can be generated, thereby greatly limiting the number of relevant, virus-related, sequences. To increase the number of reads mapping to viral RNA, several methods have been employed to either enrich non-ribosomal RNA or remove unwanted rRNA sequences. Enrichment methods include poly-A selection [TruSeq mRNA (Illumina)], Single Primer Isothermal Amplificaiton (SPIA) (Ovation^Ⓡ^ RNA Amplification System, NuGen) and Not so random (NSR) sequencing (Universal Prokaryotic RNA-Seq, NuGen). For a detailed summary of enrichment methods, refer to Table 2.

**Table 2:** Comparative studies of non-rRNA enrichment or rRNA depletion.

**Paper**	**Matrix**	**Target**	**Method**	**Main findings**
Adiconis et al., 2013	Human chronic myeloid leukemia cell line K-562, intact and degraded	tRNA	DSN-lite	RNase H lowest reads mapped to rRNA
			RNase H	
			Ribo-Zero	RNase H best for low quality
			NuGEN	
			SMART	Ribo-Zero expensive alternative to RNase H
			TruSeq mRNA	
Fauver et al., 2019^*^	Aedes, Anopheles, and Culex	Virus RNA and host transcriptome	In house PDD	Increased reads viruses and host mRNA
				Detected more intra host variants
Hasing et al., 2016	Stool samples	Norovirus	Ribo-Zero^Ⓡ^ bacterial kit (Epicentre)—subtractive hybridization	0.01 to 1.9 % of NoV reads in clinical samples
Hedegaard et al., 2014	FFPE cancer and normal tissue samples	Host transciptome	Ribo-Zero Magnetic Gold Kit—subtractive hybridization	Insufficient mixing of removal reaction lead to formation of vesicles and excessive rRNA reads
				Freezing magnetic rRNA probe binding beads decreased capture
				Strand-specific seq differentiated rRNA contamination and contamination with rRNA probes
Herbert et al., 2018	Universal Human Reference RNA (UHR) from Agilent and degraded UHR	Host transcriptome	Illumina's RiboZero	RNase H treatment or ZapR more consistent results than subtractive hybridization
			Qiagen GeneRead rRNA depletion	All kits showed strong strand bias
			Lexogen RiboCop	
			NEBNext rRNA depletion	Bias toward shorter transcripts
			Kapa RiboErase	
			Takara/Clontech's RiboGone	Kapa RiboErase kit strong bias GC transcripts
			Takara/Clontech SMARTer Pico kit	
Huang et al., 2020	Bacterial cell culture supernatant	Bacterial gene expression	In house PDD + RNase H (NEB)	Longer incubation times improved depletion efficiency for Hybridase RNase H
			In house PDD + Hybridase RNase H	Hybridase RNase H enzyme outperformed the NEB RNase H enzyme at all ratios
			Ribo-Zero^Ⓡ^ bacterial kit (Epicentre)—subtractive hybridization	Similar or outperformed Ribo Zero
Lahens et al., 2014	Plasmid from Mammalian Gene Collection (MGC)	Host transcriptome	Ribo-Zero Magnetic Gold Kit—subtractive hybridization	rRNA depletion most significant variability in coverage
Manso et al., 2013	Blood	Virus RNA	RiboErase kit (KAPA Biosystems)	rRNA-depleted panel 40- to 150-fold higher.
				Genome coverage and median depth values higher
Marston et al., 2013	Virus cell culture supernatant samples	Virus RNA (lyssavirus)	Terminator^TM^ 5′-Phosphate-Dependent Exonuclease (Lucigen)	Additional preparation not rewarded with significant improvement of viral-specific reads or read depth
Matranga et al., 2014	Mastomys natalensis and human blood and serum	Viral RNA (Lassa and Ebola virus)	In house PDD	Strand-specific sequencing discriminates viral genome and complementary RNA intermediates
				rRNA samples extracted with kits containing poly(rA) RNA contaminated high-molecular-weight by products
Palomares et al., 2019	UHR (Takara Bio/Clontech + mixture 23 human tissues	Host transcriptome	TruSeq mRNA	polyA selection more efficient than subtractive hybridization
	First Choice Human Brain reference RNA (Ambion) + pool human brain tissues		TruSeq stranded mRNA	
			TruSeq stranded total RNA Gold	
			stranded SMARTer technology (Takara Bio/Clontech) +RiboZero Gold kit (Illumina)	rRNA depletion negative effect sequencing quality
			stranded SMARTer technology + RiboGone Mammalian kit (Takara Bio/Clontech)	
			SMARTer Ultra Low technology (Takara Bio/Clontech)	
Pecman et al., 2017	Various plants with and without confirmed infection	ssRNA + virus	TailorMix miRNA Sample Preparation Kit V2 (SeqMatic LLC, USA)	Higher recovery of virus reads for ssDNA viruses and viroids when using TailorMix miRNA Sample Preparation Kit V2
		ssRNA—virus	ScriptSeq^TM^ Complete Kit (plant leaf) (Illumina, USA)—subtractive hybridization	Higher recovery of virus reads for linear RNA viruses with rRNA depletion
		dsDNA virus		rRNA depleted total RNA generated longer contigs, covering greater fractions of viral genomes
		viroids		
Petrova et al., 2017	Biofilm (*Pseudomonas aeruginosa*)	Bacterial biofilm transcriptomics	Ribo-Zero rRNA Removal Kit (Bacteria)	Ribo-Zero kit highest degree of rRNA depletion, increase in non-rRNA transcripts and increased depth of coverage.
			Ambion MICROBExpress^TM^ Bacterial mRNA Enrichment Kit (Life Technologies)	rRNA removal enhanced detection of low abundance transcripts
			RiboMinus Transcriptome Isolation Kit, Bacteria).	
Rosseel et al., 2015^^†^^	Spiked leghorn chicken serum and tissue	Virus RNA (Newcastle disease virus)	Ribo-Zero Magnetic Gold Epidemiology kit (Epicentre Technologies)	rRNA depletion of tissue RNA increased numbers of NDV reads and genome coverage but not in serum
			ScriptSeq Complete Gold Epidemiology Kit (Epicentre Technologies)	
Shanker et al., 2015	HUR total RNA [Clontech] spiked in with ERCC control mix	Host transriptome	TruSeq V2 RNA Illumina	Ribodepletion provides equivalent or superior quantitative expression data compared to the tested polyA approaches
			Ribo-Zero Gold Kit for human, mouse, or rat (Epicentre)	
			SMARTer Ultra Low RNASeq System	
			SuperAmp (R&D Systems, Minneapolis, MN, USA)	Kit protocols robust enough to perform outside of the manufacturer's recommendations
			Ovation RNA-Seq System V2 (NuGEN Technologies Incorporated)	
			SeqPlex RNA (Sigma-Aldrich; R&D Systems)	
Wongsurawat et al., 2019	Cell culture supernatant	Virus RNA	Ribo-Zero Gold kit (Illumina)	160-fold increase in proportion of viral RNA reads/host reads
				High proportion of low-quality sequences compared to non-rRNA depleted samples
Zhao et al., 2018b	Human blood and colon tissue samples	Host transcriptome	Ribo-Zero rRNA Removal kit	rRNA depletion captured more unique transcriptome features
			TruSeq stranded mRNA	polyA+ selection higher exonic coverage and accuracy of gene quantification.

* *Environmental application*,

† *Veterinary application*.

During poly-A selection, protein-coding polyadenylated RNA are captured by oligo (dT) primers attached to magnetic beads to isolate RNA. Non-polyadenylated RNA, such as rRNA, are not captured. This approach does result in a strong bias toward the 3' end of RNA targets, though this bias is alleviated by the reduced sequencing depth required to obtain high quality viral reads (Sun et al., 2013; Fonager et al., 2017; Zhao et al., 2018b).

NSR sequencing uses hexamer or heptamer primers that bind to non-rRNA target during reverse transcription (RT) (Figure 2). Several versions of NSR primer panels have been published for various applications (Endoh et al., 2005; Pyrc et al., 2007; de Vries et al., 2011; Manso et al., 2013; Xu et al., 2014; Shanker et al., 2015). NSR sequencing works well with partially degraded or low-input samples but exhibits off-target priming and is species dependent (Armour et al., 2009). During SPIA, a set of reactions occur in which a DNA/RNA chimeric primer binds the complementary sequence and is extended by a DNA polymerase at a constant temperature. Once extension of the primer is complete, the RNA is cleaved and digested by RNase H and the entire process is repeated, producing multiple copies of the amplification product (Figure 2). SPIA requires high input amounts of total RNA, which can be challenging when dealing with clinical samples but has been successfully used for detection of bovine coronavirus (Hrdlickova et al., 2017; Myrmel et al., 2017).

##### 2.2.3.2. rRNA Depletion

**Figure 3:**
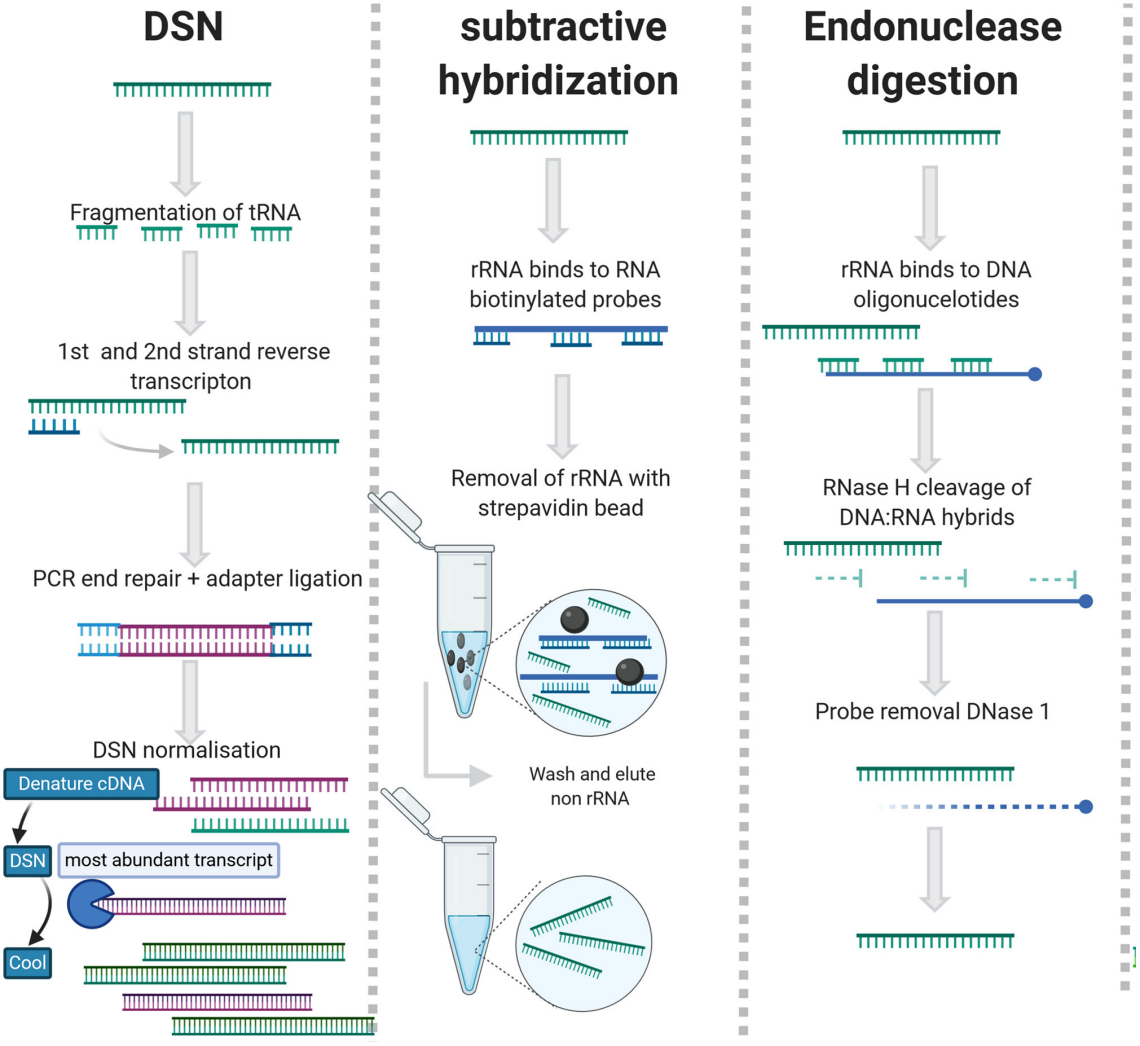
rRNA depletion methods for agnostic sequencing.

As an alternative to enrichment methods, rRNA can be removed using subtractive hybridization [Ribo-Zero (Illumina)], exonuclease digestion [MICROBExpress (Ambion)], endonuclease digestion (RNase H), or duplex specific nuclease (DSN)/Probe directed degradation (PDD). An overview of the rRNA depletion methods can be viewed in Figure 3 and detailed summary of studies applied rRNA depletion in viral metagenomic studies is included in Table 2.

DSN generates rRNA depleted libraries by employing the C0t-kinetics-based normalization method to deplete abundant sequences that re-anneal quickly, ergo highly abundant rRNAs and tRNAs (Xiao et al., 2013) (Figure 3). The DSN method works with lower concentrations of RNA and partially degraded rRNA but requires a longer time to prepare libraries (Yi et al., 2011; Qiu et al., 2015). During PDD, rRNAs are targeted by anti-sense DNA oligos and digested by RNase H/DSN (Morlan et al., 2012; Kim et al., 2019) (Figure 3). This requires saturation of rRNA with contiguous oligonucleotide and is slightly less efficient than subtractive hybridization (Archer et al., 2014). The advantage of this approach is that the probes are essentially reverse primers so they are cheap and easy to design (Fauver et al., 2019). DSN has been applied successfully for the sequencing of RNA viruses in complex matrices (Schuh et al., 2020; Zhou et al., 2020). For subtractive hybridization, unwanted rRNAs/cDNAs are hybridized to biotinylated DNA or Locked nucleic acid (LNA) probes and depleted with streptavidin beads (Briese et al., 2015; Culviner et al., 2020).

## 3. Steps in Procedure

### 3.1. RNA Extraction

There are three common methods used for RNA extraction: organic extraction using Acid guanidinium thiocyanate phenol chloroform (AGPC), Silica membrane based spin column technology (SMSC), and silica coated Magnetic beads (MB). Due to the physio-chemical differences between these three extraction methods, the yield, purity, and specificity of the RNA obtained varies and this can have downstream impacts.

AGPC extraction dissolves cell/viral components and maintains the integrity of RNA, due to the denaturing activity of phenol and guanidine thiocyanate with respect to RNases (Le et al., 2018). The addition of chloroform or a chloroform alternative, followed by centrifugation, separates RNA from DNA, proteins, lipids and insoluble matter (Le et al., 2018). However, RNA isolated by this method is often contaminated with protein, cellular materials and organic solvents such as phenol-chloroform, salts and ethanol (Tavares et al., 2011). In addition, the phenol may render the RNA incompatible with downstream applications. SMSC and MB based RNA isolation systems do not require the use of organic solvents, are relatively simple, efficient, low cost, and yield total intact RNA with low levels of contamination from proteins and other cellular materials. However, these methods can often result in significant levels of genomic DNA contamination, an important consideration with respect to sequencing of viral RNA (Tavares et al., 2011).

MB can be coated with silica, oligo(dT) or specific capture probes. Silica coated MB non-selectively bind nucleic acids in the presence of chaotropic salts *via* electrostatic interactions. The silica-coated beads are most suitable for applications that require nucleic acids other than mRNA while the oligo (dT) beads are best-suited for mRNA targets that are polyadenylated. Specific capture-based systems are best suited for applications that do not tolerate high concentration of non-target nucleic acids (Adams et al., 2015). There is limited information available with respect to the impact of RNA concentration and extraction on sequencing results. Those that are available are summarized in Table 3. Of interest to this review are results from Hjelmsø et al. (2017), comparing the impact of sewage concentration and RNA extraction methods on viral sewage metagenomics. Hjelmsø and colleagues found that (i) highest viral specificity was obtained using Polyethylene glycol (PEG) concentration (ii) Nucleospin RNA XS generated the highest read count for RNA viruses (norovirus, rotavirus and Hepatitis A and E virus) and (iii) viral richness is strongly impacted by extraction method. In a similar fashion, Strubbia et al. (2019a) found that PEG extraction resulted in longer contigs and detection of other viruses in sewage samples. However, this was outperformed by an alternative method which applied sodium pyrophosphate combined with a sonication step prior to PEG concentration. This method successfully generated norovirus reads from both sewage and oyster digestive tissue (Strubbia et al., 2019b). Considering non-viral studies outlined in Table 3, the evidence for improved RNA Integrity number (RIN) values and higher concentrations of RNA using organic extractions is mixed, with no clear trend in either direction.

**Table 3:** Comparison of RNA extraction methods used for HTS.

**Paper**	**Target RNA**	**Extraction kit/platfo**	**Type of extraction**	**DNase**	**RIN**	**Best performing extraction method**
Aarem et al., 2016	microRNA (6 generic, 13 specific)	MagMAX^TM^ for Stabilized Blood Tubes RNA Isolation Kit, compatible with Tempus^TM^ Blood RNA Tubes manual and semi-manual, Preserved Blood RNA Purification Kit I (for use with Tempus Blood RNA Tubes), Tempus^TM^ Spin RNA Isolation Kit,Tempus^TM^ 6-Port RNA Isolation Kit	Magnetic bead-based RNA purification system, spin column chromatography	TURBO DNase with MagMAX	7.88–7.93 average for adult and cord sample	No significant differences
Ahmed et al., 2019		Cetyl trimethylammonium bromide (CTAB) with additional wash steps, potassium acetate, and lithium chloride for precipitation	Organic extraction (CTAB)		7.3–8.8	Modified CTAB
Asai et al., 2015	tRNA	TRIzol^TM^ Reagent, Qiagen RNeasy Micro Kit, Aurum Total RNA Mini Kit	Organic extraction, Silica Spin Filter Columns, Spin- or vacuum-mediated silica binding	Included in Qiagen and Aurum kits	3.9–8.9	Qiagen RNeasy Micro Kit
Di et al., 2019^^†^^	Highly pathogenic avian influenza (HPAI) H5N1	1: MagNA Pure compact RNA isolation-Lysis/Binding buffer (Roche)+ MagNA Pure compact RNA isolation kit [RNA-Tissue-V3-1 protocol (Roche)]. 2: Buffer RLT (QIAgen) containing 1%-mercaptoethanol+ RNeasy mini kit. 3: Buffer AVL (QIAgen) + QIAamp viral RNA mini kit (QIAgen). 4: TRIzol reagent (Invitrogen) + TRIzol/chloroform (Invitrogen).	MagNA Pure Magnetic Glass Particle Technology, Silica Spin Filter Column, Organic extraction	Turbo DNA-free kit (Ambion)		MagNA Pure compact RNA isolation-Lysis/Binding buffer (Roche)+ MagNA Pure compact RNA isolation kit [RNA-Tissue-V3-1 protocol (Roche)].
Garcia-Nogales et al., 2010^*^	16S/23s rRNA	RiboPure^TM^ Bacteria Kit (Ambion), RNeasy Protect Bacteria Mini kit (Qiagen), NucliSENS^Ⓡ^ miniMAG^Ⓡ^ (Biomerieux) + mechanical disruption cycle, TRIzol^Ⓡ^ Max Bacterial (Invitrogen) + mechanical disruption cycle	Organic Extraction +Spin column, Silica-coated magnetic beads (BOOM technology), Organic extraction + mechanical disruption			RNeasy Protect Bacteria Mini kit from QIAGEN
Guichet et al., 2018	HIV RNA	NucliSens EasyMag (Biomérieux, Marcy-l'Etoile, France), NucliSens EasyMag + free virus elution method, m2000sp method (Abbott Molecular, IL, USA)	Silica-coated magnetic beads (BOOM technology), Silica-coated magnetic beads (BOOM technology) +FVE	Turbo DNase-free (Ambion by Life Technologies, CA, USA) and HL dsDNase (“Heat and Run” gDNA removal kit, TATAA Biocenter AB, Sweden)		NucliSens EasyMag + free virus elution method
Hedegaard et al., 2014		QIAsymphony RNA Mini Kit and performed on QiaSymphony robot, Nucleospin FFPE RNA/DNA kit (Machery-Nagel), RecoverAll Total Nucleic Acid Isolation Kit for FFPE (Ambion), purification with: miRNeasy FFPE (QIAGEN), Nucleospin FFPE RNA (Machery-Nagel) and ExpressArt FFPE RNAready (Amp Tec) kits	Silica-coated magnetic beads, silica membrane technology, Spin Column (Glass Fiber Filter)	DNase I and the other was additionally treated with Exonulcease I		miRNeasy FFPE (QIAGEN) and ExpressArt FFPE RNAready (Amp Tec)
Hjelmsø et al., 2017^*^	Viral DNA/RNA, MCO RNA	Nucleospin RNA XS, QIAamp Viral RNA Mini Kit, NucliSENS^Ⓡ^ miniMAG^Ⓡ^, or PowerViral^Ⓡ^ Environmental RNA/DNA Isolation Kit	Spin Column (Glass Fiber Filter), spin column-based RNA purification, Silica-coated magnetic beads (BOOM technology), Silica Spin Filter Columns with chemical lysis	OmniCleave endonuclease (Epicentre, Wisconsin, USA). Further purified by extraction using a 1:1 mixture of chloroform-butanol		Nucleospin RNA XS
Le et al., 2018^*^	HAV, NoV GI, NoV GII	Nuclisens EasyMag (Biomérieux, Marcy-l'Etoile, France), Trizol (Invitrogen) + Purelink mini RNA kit+CTAB+LiCl precipitation	Silica-coated magnetic beads (BOOM technology), Organic extraction, Organic extraction (CTAB)			Trizol, PureLink RNA Mini Kit, followed by Cetyltrimethylammonium bromide (CTAB) treatment and LiCl precipitation
Li et al., 2015	DNA and RNA viruses	QIAamp Viral RNA Mini Kit (Qiagen, Hilden, Germany), Maxwell 16 Viral Total Nucleic Acid Purification Kit (Promega, Madison, WI, USA), and Trizol Reagent (Life Technologies, Grand Island, NY, USA)	Silica Spin Filter Columns, chemical lysis+ Magnetic bead-based RNA purification system, GTPC	Turbo DNase (Ambion, Life Technologies, Grand Island, NY, USA), 3U Baseline-ZERO (Epicentre, Chicago, IL, USA)		QIAamp Viral RNA Mini Kit (Qiagen, Hilden, Germany)
Marston et al., 2013	Lyssavirus RNA	TRIzol +PEG, RNeasy plus mini kit	Organic extraction, Silica Spin Filter Columns	RNeasy plus mini kit		TRIzol +PEG
Pauly et al., 2019	HAV, HBV, HCV, HDV, and HEV.	MagNA Pure Compact Nucleic Acid Isolation Kit I, MagNA Pure LC 2.0 Total Nucleic Acid	MagNA Pure Magnetic Glass Particle Technology, magnetic-bead technology			MagNA Pure ure LC 2.0 for ssRNA best
		Isolation Kit, MagNA Pure LC 2.0 Total Nucleic Acid Kit - High Performance, MagNA Pure 96 DNA and				
		Viral NA Small Volume Kit				
Schwochow et al., 2012	miRNA	RiboPure,RNeasy^Ⓡ^, PAXgene^TM^, TRIzol^Ⓡ^ LS,LeukoLOCK^TM^	Spin column + organic extraction, Silica Spin Filter Columns, organic extraction, Leukocyte Capture Filter+Magnetic Bead	rDNaseI (Ambion)	4.6–7.7	LeukoLOCK^TM^ filter system
Strubbia et al., 2019a^*^	norovirus	PEG, PEG +sodium chloride at pH 3, PGM capture+ PEG, NucliSens kit (bioMerieux), Zymo-spin column (RNA Clean & Concentrator, Zymo Research, Irvine, USA) for all	Organic extraction, Silica-coated magnetic beads (BOOM technology), Silica Spin filter column,	Turbo DNAse		PEG samples had longer contigs, no clear optimal extraction process
Strubbia et al., 2019b^*^	norovirus	PEG, Pyro-PEG, PK-PEG, NucliSens kit (bioMérieux)	PEG+ Silica-coated magnetic beads (BOOM technology), sodium pyrophosphate decahydrate +sonication+PEG+ Silica-coated magnetic beads, Proteinase K lysis +Silica-coated magnetic beads	TURBOTM DNase		Pyro-PEG sewage, PK-PEG shellfish
Sultan et al., 2014	mRNA	RNeasy preparation method (Qiagen), TRIzol PARIS (Life Technologies)	Silica Spin Filter Columns, Organic extraction	TURBO DNA-free^TM^ (Life Technologies, #AM1907)		TRIzol
Tavares et al., 2011	28s/18s rRNA	TRIzol^Ⓡ^ Plus RNA Purification System (Invitrogen), E.Z.N.A.^TM^ Total RNA kit II (Omega Bio-Tek), AxyPrep Multisource Total RNA Miniprep, RNeasy^Ⓡ^ Mini, EasySpin and Illustra RNAspin Mini RNA Isolation	Organic extraction, Silica Spin Filter Columns	Turbo^TM^ DNase I (Ambion)		AxyPrep Multisource Total RNA Miniprep kit
Wong et al., 2019	miRNA	MagnaZol (Bioo Scientific) or miRNeasy (QIAGEN)	Magnetic bead-based RNA purification system, Silica Spin Filter Columns			MagnaZol RNA

* *Environmental application*,

† *Veterinary application*.

### 3.2. cDNA Generation

#### 3.2.1. Impact of Reverse Transcriptase Enzyme

Both the efficiency of reverse transcription and fidelity is important for the detection of virus quasispecies present at low abundances and the identification of SNV. To date only two studies have investigated reverse transcriptase (RT) fidelity impact on next generation sequencing results (Cholet et al., 2020; Zucha et al., 2020). In order to demonstrate the impact that RT has on the quality of cDNA synthesized, this section includes earlier studies where the focus is on cDNA yield/RT enzyme efficiency for quantitative Reverse Transcription Polymerase Chain Reaction (qRT-PCR) applications (see Table 4). Whilst the focus in these studies is primarily the efficiency in terms of yield, this is still an important consideration for shotgun metagenomics, where relative abundance of viral targets could be interpreted as prevalence, and for the detection of RNA templates present at low concentrations.

**Table 4:** Literature investigating the impact of RT enzyme on cDNA yield.

**Paper**	**RT enzymes**	**Main findings**
Levesque-Sergerie et al., 2007	SuperScript II, SensiScript, PowerScript and OmniScript	RT influenced by concentration of background RNA
		Low abundance transcripts yielded more cDNA when using SuperScript II
Lindén et al., 2012	SuperScript II, AMV-RT, Transcriptor RT, M-MuLV RT, M-MLV RT, Omniscript, DyNAmo, Statascript	Covariances of the RT efficiency were driven by target gene or total RNA concentration
		Enzyme differences less important than diversity in gene-specific RT reproducibility
Bustin et al., 2015	iScript, Vilo, Grandscript, Readyscript, Primescript, and Tetro RT enzyme	Variation observed was greater between RT enzymes, than between technical replicates
Miranda and Steward, 2017	SuperScript II, SuperScript III	Increasing background RNA and primer concentrations increased cDNA yield, but benefit of background RNA was source dependent.
Schwaber et al., 2019	SuperScript III VILO Kit, Superscript II and Protoscript	Optimum RT conditions are transcript specific and driven by RNA concentration

#### 3.2.2. Priming Strategy

Random primers are oligonucleotides with random base sequences widely applied during RT as described in the section on SISPA (2.2.1). As noted above, they are often six nucleotides long and are usually referred to as random hexamers, N6, or dN6. Due to their random binding, they can potentially anneal to any RNA species in the sample. Therefore, these primers may be considered for reverse transcription of RNAs without poly(A) tails, degraded RNA and RNA with known secondary structures. Some random primer sets have been constructed with viral genomes in mind, preferentially priming viral RNA over ribosomal RNA (Endoh et al., 2005; Strubbia et al., 2020).

Oligo(dT) primers consist of a stretch of 12–18 deoxythymidines that anneal to poly(A) tails of eukaryotic mRNAs, which make up only 1–5% of total RNA. Oligo(dT) primers target polyadenylated RNAs, whereas random sequence primers target all RNAs including the abundant rRNA fraction. Mixtures of random hexamers with oligo(dT) are predominantly used in qRT-PCR to maximize yield. Oligo(dT) priming has also been applied as a viral RNA enrichment method as outlined earlier 2.2.3.

Gene-specific primers offer the most specific priming in RT (Miranda and Steward, 2017). These primers are designed based on known sequences of the target RNA, requiring prior knowledge. Since the primers bind to specific RNA sequences, a new set of gene-specific primers is needed for each target RNA. Primers that are specific to a viral genome also efficiently eliminate the influence of ribosomal RNAs.

#### 3.2.3. Norovirus a Case Study of Various RT Approaches for Viral HTS

Strubbia et al. (2020) reviewed three sets of hexamers, those from Endoh et al. (2005), an updated version of this hexamer panel (I-HD), including a probe to reduce host rRNA from oysters, and random hexamers. The I-HD panel resulted in lower read numbers aligning to Mollusc and other Eukaryote genomes. Furthermore, the number of reads targeting virus sequences was higher compared to the random set. Conversely, random hexamers produced more reads aligning to HuNoV than the custom panel and those from Endoh et al. (2005). Random hexamers transcribed HuNoV sequences more efficiently and produced longer contigs, allowing HuNoV genotype identification.

In Table 5 below, the variety of reverse transcriptase enzymes and priming strategies applied in norovirus HTS studies can be seen. SuperScript II, SuperScript III, and High Capacity cDNA RT were commonly used for cDNA synthesis, whilst a balance of random hexamers and oligo(dT) priming strategies were popular. As most publications have not assessed the yield/fidelity post cDNA synthesis, it is not possible to compare these publications based on the RT experimental design. Strubbia et al. (2020) demonstrate that priming strategy for norovirus alters the contig length, which is important for genotypic characterization, but this study did not include oligo(dT)s, or a comparison of RT enzymes.

**Table 5:** RT enzyme and priming strategies used for norovirus NGS studies.

**Paper**	**Reverse transciptase enzymes**	**Priming strategy**
Bartsch et al., 2018^*^	Not provided	Not provided
Bavelaar et al., 2015	REPLI-g sc Polymerase	Random hexamers and oligo dT primers
Boonchan et al., 2017	Qiagen One Step RT PCR enzyme (Sensiscript and Omniscript Reverse Transcriptases, HotStarTaq DNA Polymerase)	Not provided
Brown et al., 2016	SuperScript III	Random hexamers
Casto et al., 2018	SuperScript III	Random hexamers
Chan et al., 2017	SuperScript III	Tagged random octamers
Chen et al., 2018	Ovation RNA sequencing (RNA-Seq) system version 2 kit (NuGen, USA)	Chimeric primer mix
Chhabra et al., 2018	Not provided	In-house degenerate primer
Cotten et al., 2014a	SuperScript III	Tiling approach custom primer panel
Cotten et al., 2014b	SuperScript III	Endoh et al. (2005) hexamers
Cuevas et al., 2016	SuperScript III, AccuScript Hi-Fi reverse transcription (Agilent)	Random hexamers, custom primer
Fonager et al., 2017	SMARTer RNA stranded sequencing kit	Oligo(dT)
Fumian et al., 2019^*^	High Capacity cDNA Reverse Transcription Kit and MultiScribe^TM^ Reverse Transcriptase (Thermo Fisher)	Random primers
Hasing et al., 2016	TruSeq RNA sample preparation kit v2 with SuperScript II	Oligo(dT)
Imamura et al., 2016a^*^	High Capacity cDNA Reverse Transcription Kit and MultiScribe^TM^ Reverse Transcriptase (Thermo Fisher)	Oligo(dT)
Imamura et al., 2016b^*^	High Capacity cDNA Reverse Transcription Kit and MultiScribe^TM^ Reverse Transcriptase (Thermo Fisher)	Oligo(dT)
Imamura et al., 2017^*^	High Capacity cDNA Reverse Transcription Kit and MultiScribe^TM^ Reverse Transcriptase (Thermo Fisher)	Oligo(dT)
Kundu et al., 2013	SuperScript III	Random hexamers
Nasheri et al., 2017	TruSeq Stranded mRNA SuperScript III	Random hexamers
Strubbia et al., 2019a^*^	SuperScript II	Random hexamers
Strubbia et al., 2019b	SuperScript II, SuperScript III	Non-ribosomal hexamers (Endoh et al., 2005), random hexamers
Strubbia et al., 2020^*^	SuperScript II	Random hexamers (New England Biolabs (NEB), USA), I-HD hexamers, non-ribosomal hexamers (Endoh et al., 2005)
Suffredini et al., 2018^*^	MyTaq^TM^ One-Step RT-PCR Kit	Target specific primers
van Beek et al., 2017	SuperScript III	Random hexamers

* *Environmental application*.

### 3.3. Amplicon Generation

For traditional amplicon sequencing, primers should target a conserved region to allow for reliable detection of the viral target. Primers should be checked against recent sequences of the target question and the PCR conditions (particularly if DNA polymerase enzyme is altered) should be optimized and validated internally. “Jackhammer” PCR allows greater room for error in this aspect, as the primers are targeted across the genome, increasing the probability of successful amplification. That said however, viral RNA is a moving target and “jackhammer” approaches require up to date sequence data to perform consistently. Aside from primer design and method validation, additional considerations given to amplicon generation in HTS protocols is the choice of DNA polymerase and associated PCR cycling conditions and cycle numbers. Amplification errors generated during PCR appear in sequencing data and contribute to false mutations that can ultimately confound genetic analysis (Potapov and Ong, 2017). Several high-fidelity polymerase enzymes are commercially available and have been assessed using a variety of targets for downstream sequencing, see Table 6. Polymerase choice impacts both occurrence and relative abundance estimates and it has been noted that DNA polymerase choice had a greater impact on correct sequence assignment than a reduction in PCR cycles (Quail et al., 2011; Brandariz-Fontes et al., 2015; Nichols et al., 2018). Target characteristics such as prevalence of GC/AT rich regions, as can occur with Hepatitis E, may require an optimized approach for PCR amplification. Additives such as Dimethyl sulphoxide (DMSO) for GC-rich templates or betaine for AT-rich templates can reduce amplification bias for such targets. Betaine may help to keep a GC-rich template single-stranded, but it may also cause premature dissociation of the newly synthesized strand from an AT-rich template, introducing knock on effects for virome analysis (Aird et al., 2011; Nichols et al., 2018). Secondary structures in templates can also bias PCR when molecules with secondary structures, such as hairpin structures common in RNA templates, bind to themselves and inhibit their own amplification. This feature has been utilized in linker-amplification shotgun library second generation sequencing methods (Angly et al., 2006).

**Table 6:** Performance of various DNA polymerases enzymes applied during targeted HTS.

**Paper**	**Target**	**Matrix**	**DNA polymerases**	**Outcome**
Aird et al., 2011	*Plasmodium falciparum*	Purified DNA extract	Phusion HF	AccuPrime Taq HiFi performed best for GC rich templates
	*Escherichia coli*		AccuPrime TaqHiFi	Thermocycler and temperature ramp rate introduce bias
	*Rhodobacter sphaeroides*			
Brandariz-Fontes et al., 2015^^†^^	Mitochondrial DNA from wolves		Phusion High Fidelity DNA Polymerase (Finnzymes)	Enzyme greater impact on the number of correct reads than other factors
			KAPA HiFiTM (Kapa Biosystems)	
			Phusion Pwo DNA Polymerase (Roche)	
			AmpliTaq Gold (Applied Biosystems)	
			i-MaxTM II DNA Polymerase (iNtRON Biotechnology)	
			Taq DNA Polymerase (Roche)	
			Velocity DNA Polymerase (Bioline)	
	MHC class I exon 3 (MHC I) in horse		HotStarTaq DNA Polymerase (Qiagen)	Phusion Pwo and Kapa HiFi worked best
			FastStart High Fidelity PCR System (Roche)	
			Biotaq (Bioline) Biotaq	
			OneTaq DNA Polymerase (New England Biolabs)	
			Vent DNA Polymerase (New England Biolabs) Vent	
			Deep Vent DNA Polymerase (New England Biolabs)	
Dabney and Meyer, 2012	Genomic DNA	Human and Neandertal samples	Herculase II Fusion	AccuPrime Pfx performed best
			Phusion Hot Start I and II with HF and GC buffers	
			Phusion High Fidelity Master Mix	
			AmpliTaq Gold	
			Platinum Taq High Fidelity	Phusion polymerases in HF buffer and AmpliTaq Gold dramatic biases
			Pfu Turbo Cx Hotstart	
			AccuPrime Pfx Polymerase	
Jia et al., 2014	BRCA1 and BRCA2 genes	human	SequalPrep polymerase (Invitrogen, Carlsbad, CA)	Coverage varied widely amongst polymerases, particularly for exon and intron regions.
			AccuPrime Taq DNA Polymerase (Invitrogen, Carlsbad, CA)	
			PrimeSTAR GXL polymerase (TaKaRa Bio, Shiga, Japan)	
			LA Taq Hot Start Version Polymerase (TaKaRa Bio, Osaka, Japan)	PrimeSTAR GXL DNA Polymerase performed best for long range PCR SNV detection
			KAPA long Range HotStart DNA polymerase (KAPA Biosystems, Wobum, MA)	
			QIAGEN LongRange PCR Polymerase (Hilden, Germany)	
Nichols et al., 2018^*^	Soil microbiome	Sedimentary DNA samples	AmpliTaq Gold, Buffer II	Qiagen Multiplex Master Mix polymerase accurately reconstructed relative abundances e, but also generated the highest error rate
			Kapa HiFi ReadyMix	
			Phusion	
			Platinum HiFi	
			Q5 2x Master Mix	
			Qiagen Multiplex Master Mix	
Quail et al., 2011	*Bordetella pertussis*	Unclear	Accuprime pfx	Kapa HiFi performed the best overall though
			Accuprime Taq Buffer I	
			Advantage HF 2	
			Ex Taq	
			Herculase II	
			iPROOF	
	*Salmonella pullorum*		ISIS	
			Kapa HiFi	
			Kapa HiFi qPCR blend	
			Kapa2G Robust Hotstart	
			Optimase	
			Pfu Turbo	
	*Staphylococcus aureus*		Pfu Ultra Hotstart	
			pfu ULTRA II fusion HS	Genome coverage using Kapa HiFi more uniform than that with Phusion, but higher error rate
			Pfx50	
			Phusion	
			Phusion Flash	
			Platinum Taq HiFi	
	*Plasmodium falciparum*		Precisor	
			Pwo master	
			Taq polymerase	
			Topo Taq HF	
			Twist Amp Basic	
Stasik et al., 2018	c.1849G > T (p.Val617Phe) mutation of the JAK2-gen		Platinum^Ⓡ^ Taq Platinum	High accuracy proofreading polymerases significantly (5-fold) reduced median per-base
			AmpliTaq Gold^Ⓡ^ PCR Gold Buffer 1x Activation	Q5 High Fidelity polymerase reduced both transition and transversion bias, mainly for T > C (25-fold), T > A and G > C (11-fold each)
			Phusion Hot Start II^Ⓡ^ Phusion HF Buffer 1x Activation	AmpliTaq Gold performed poorly
			Q5^Ⓡ^ High-Fidelity Q5 Reaction Buffer 1x Activation	

† *Veterinary application*,

* *Environmental application*.

### 3.4. Fragmentation

Following poly(A) + selection or rRNA depletion, RNA samples are fragmentated to a certain size range, owing to the limitations in the read length of many HTS platforms (Hrdlickova et al., 2017). RNAs can be fragmented with alkaline solutions, solutions with divalent cations, such Mg++, Zn++, or enzymes, such RNase III. Fragmentation with alkaline solutions or divalent cations is typically carried out at an elevated temperature to mitigate the effect of RNA structure on fragmentation (Hrdlickova et al., 2017).

Alternative RNA-Seq library preparations have been suggested to overcome fragmentation bias, including ClickSeq technology and the incorporation of barcoded non-ribosomal hexanucleotide primers during reverse transcription (Routh et al., 2015; Jaworski and Routh, 2017; Wang et al., 2017). In ClickSeq, reverse transcription (RT) reactions are performed with 3'-azido-nucleotides (AzNTPs). AzNTPs are chain-terminators that stochastically terminate cDNA synthesis as determined by AzNTPs:dNTPs. Following chain termination, single-stranded cDNA fragments are generated with an azido-group at their 3' ends. 3'-azido-blocked cDNA molecules can be purified and “click-ligated” to 5' alkyne-modified DNA adaptors *via* copper-catalysed azide-alkyne cycloaddition (CuAAC). The products of the ClickSeq reaction can be amplified using PCR to generate a cDNA sequencing library. Viral RNAs and mRNA using ClickSeq produced unbiased HTS libraries with low error-rates compared to standard methods (Routh et al., 2015; Jaworski and Routh, 2017).

Alternatively, intact RNAs can be reverse transcribed, and full-length cDNA can be fragmented. DNA is fragmented using either mechanical methods (e.g., nebulization and ultrasonication shearing) or enzymatic digestion. Nebulization involves directing compressed nitrogen or air forces into a DNA sample repeatedly through a small hole, producing mechanically sheared random fragments, leading to a heterogeneous mix of double-stranded DNA molecules containing 3'- or 5' overhangs as well as blunt ends (Knierim et al., 2011). During sonication, DNA is subjected to ultrasonic waves, whose vibrations produce gaseous cavitations in the liquid that shear or break high molecular weight DNA molecules through resonance vibration. Enzymatic digestion of DNA can take many forms, dependent on the library sequencing kit chosen. In general, the fragmented DNA is ligated at both blunt ends of each fragment with specific adaptors, using a transposon-based, tagmentation enzyme. These ligated sites later serve as primer-binding sites for amplification (Poptsova et al., 2014; Hrdlickova et al., 2017). A key issue with fragmentation is that the shear time is difficult to control because DNA or RNA originate from samples with different viral RNA abundance and this treatment may increase the occurrence of artifactual recombination.

### 3.5. Quality Control

Unlike RT-PCR, that is subject to Minimum Information for Publication of Quantiative Real-Time PCR Experiments (MIQE) guidelines, HTS/RNA-Seq has been to slow to include extensive controls, as per other molecular methods (Bustin et al., 2009, 2010). Issues such as contaminant RNA, cross-contamination and human error can be managed by robust experiment design that includes a variety of control samples and quality check points. Human error is unavoidable and 2–3% of samples were estimated to be mis-labeled or mis-pipetted in the Sequencing Quality Contro project (SEQC) (Qing et al., 2013). Given the observation of batch effects across studies; randomization of samples and treatment groups is pivotal and in part helps to circumvent handler bias (Qing et al., 2013; Miller et al., 2016; Eisenhofer et al., 2019).

Whole process negative controls and non-template controls can be included at sample preparation/extraction and library preparation stages. Furthermore, negative controls serve to demonstrate that the method in question does not generate false positives. While there are issues with running blank samples on some HTS platforms, negative samples can be spiked with a unique oligonucleotide to overcome primer-dimer formation issues, similar to internal process controls used in qRT-PCR assays. Cross-contamination can create “batch effects” due to the transfer of sample RNA, barcodes, or amplicons from neighboring wells or tubes. By including negative controls (extraction and library preparation) and comparing controls to biological samples post sequencing, cross-contamination can be identified, thereby aiding the interpretation of sequencing results. Strand specific sequencing can be used to identify the source of contamination during subtractive hybridization or viral genome vs. complementary RNA intermediates (Hedegaard et al., 2014; Matranga et al., 2014). Notably, the use of non-redundant dual indexing prevents index swapping during sequencing, which otherwise can contaminate up to 6% of samples (Costello et al., 2018; Du et al., 2020). Certain sequencing platforms also require maintenance washes between runs to reduce the likelihood of run-to-run cross-contamination.

While a variety of commercial positive sample controls are available, they are not always suitable as external/internal quality controls for HTS of viral RNA. Positive controls available include RNA oligonucleotides, mock virome communities (virus or nucleic acid), Spike in RNA variants (SIRVs) and External RNA Control Consortium (ERCC). RNA oligonucleotides, SIRVs and ERCC samples can be applied as internal controls, spiked into each sample, including the whole process negative control. RNA oligonucleotides and ERCC can be used to assess sample inhibition, which is important to consider in complex matrices, as well as confirm method specificity (Miller et al., 2016; Bal et al., 2018). However, Munro et al. (2014), Qing et al. (2013), and Risso et al. (2014) determined that while ERCC controls could be used as batch controls, they exhibited strong protocol dependent bias and a high degree of variation. SIRVs have been used in previous studies to assess the accuracy of SNV calling in transcriptomic bioinformatic pipelines, though these may not work for SNV detection in RNA viruses. Furthermore, the use of spike in controls assumes that technical effects impact spike-ins and target sequences in the same way. If library preparation steps impact spike-in and target read counts differently, then normalization or inhibition based on the spike-ins may be incorrectly assessed. Mock virome samples or RNA oligonucleotides can be used in a serial dilution to determine limit of detection or false discovery rate and, in the case of mock viromes, demonstrate that a variety of RNA viral families can be sequenced. To date, mock virome controls have not yet been applied in HTS of RNA viruses in environmental samples.

## 4. Discussion

### 4.1. Sample Contamination

The importance of negative controls in any molecular work, but particularly a method as sensitive as HTS/RNA-Seq, has been emphasized again and again. Multiple studies have been published noting contaminating taxa, likely from reagents (kitome), common environmental taxa introduced through cross-contamination (Salter et al., 2014; Glassing et al., 2016; Bal et al., 2018; Leon et al., 2018) and possible cross contamination (Strubbia et al., 2020). Moreover, the discovery of bacterial reads in cell line data processed using poly-A selection demonstrates that downstream contamination is a source of bacterial reads (Strong et al., 2014). Contamination can also originate from staff, plastic consumables, nucleic acid extraction kits and platforms and laboratory reagents, therefore controls should address these sources as outlined earlier. Negative controls should be compared to biological samples in the final raw sequencing reads. There is much debate as to whether or not contaminating taxa be removed from biological samples but this has been applied in various pipelines (Davis et al., 2018; Leon et al., 2018; Palmer et al., 2018). An additional pre-processing step that has been proposed is to use predictive modeling to identify putative contaminants (Risso et al., 2014; Eisenhofer et al., 2019).

### 4.2. Low Target Abundance (Viral RNA)

High mutation rate and antigenic drift of most single stranded RNA (ssRNA) viruses, makes it difficult to design reasonably sized CBPH panels that capture species diversity, while also being affordable and technically feasible (Duffy, 2018; Peck and Lauring, 2018). It must be noted though that CBPH resulted in significantly greater genome coverage, % of viral reads and depth of coverage in all studies listed in Table 1 compared to shotgun metagenomics. Whilst some cost comparisons suggest that amplicon “jackhammer” approaches are a similar cost, there is to date only one amplicon vs CBPH comparison study. It was determined that amplicon sequencing had greater on target reads, though CBPH demonstrated a significantly higher standard deviation of genome coverage a more accurate depiction of SNVs (Samorodnitsky et al., 2015). Furthermore, Nasir et al. (2020) noted that CBPH provided an advantage over amplification based protocols such as tiling amplicon approaches due to the absence of amplification artifacts.

Applications of SISPA in the veterinary field has permitted first-time detection or detection of new variants of Newcastle disease virus, Schmallenberg virus, Hantaviruses, and enterovirus C104. However, based on the comparative studies and field work applications of SISPA, it appears that its application is best placed for fieldwork, where speed rather than accuracy is the objective. Follow-up direct sequencing or targeted amplicon sequencing should be used to verify suspected SNVs. There are very few publications applying RCA-HTS to RNA templates and this is likely due to the challenge of working with samples containing abundant background RNA and low target RNA concentrations. While RCA-HTS is the least error prone target amplification approach, it is not suitable in its current format for application to low abundance RNA samples and better suited to studies involving cell culture work.

Various findings from comparative rRNA depletion/enrichment studies found that while rRNA depletion resulted in increased target reads, coverage depth and detection of intra host variants, it also increased the proportion of low-quality reads obtained (Adiconis et al., 2013). PDD incorporating RNaseH provided superior or more consistent results at lower costs, compared to Ribo-Zero/subtractive hybridization (Herbert et al., 2018; Huang et al., 2020). All depletion methods show both strand specific bias as well as a bias toward shorter transcripts (Pecman et al., 2017; Herbert et al., 2018). Pecman et al. (2017) found that rRNA depletion methods worked better for ssRNA viruses than dsDNA viruses. The limitation to these studies is that most focus on commercially available subtractive hybridization kits (see Table 2 for a more in-depth overview of the aforementioned studies). Furthermore, viral RNA is rarely the target, with the host transcriptome more typically the focus.

In terms of recommendations for agnostic sequencing, PDD is more robust and flexible in terms of host rRNA and works better with degraded samples, however there may be issues for low concentration targets, in which cases NSR is a viable alternative. SISPA and SPIA require high input concentrations of RNA and are likely to be unsuitable for samples containing low abundance of specific RNA viral targets. For low concentration targets with a poly-A tail, evidence from transcriptomics indicates that poly-A capture outperforms subtractive hybridization. Choices for targeted sequencing heavily depend on the research question. While CBPH is more expensive, it is a more suitable choice for the detection of SNVs than a “jackhammer” approach. Amplicon sequencing is suitable for well-characterized viruses, with robust PCR assays, where the purpose is genotypic characterization.

### 4.3. Bias

#### 4.3.1. Nucleic Acid Extraction Stage

Virus-specific approaches increase the chance to detect less abundant species through HTS. The quality of a HTS run has both cost and time implications, and greater viral specificity can reduce the time required for bioinformatics analyses (Hjelmsø et al., 2017). Purification steps during concentration and extraction may not increase viral RNA, but the elimination of background nucleic acids could increase the ratio of viral reads and the quality of contigs obtained (Strubbia et al., 2019b). Therefore, choice of concentration, RNA isolation/extraction and purification steps are influential in determining the quality of RNA obtained and subsequent HTS outputs. In general, AGPC methods result in better quality RNA, however the compromise is often lower concentrations of RNA. While this may not concern studies working with concentrated clinical samples, complex samples such as stool, soil or certain food matrices pose a greater challenge. In these cases, SMSC and MB methods work best for samples, containing low concentrations of viral RNA or in complex samples with high levels of background RNA. Downstream purification (DNase step, spin column purification, ethanol precipitation) of the RNA extracts may be required as SMSC/MB can carry through genomic DNA.

#### 4.3.2. cDNA Generation Stage

Overall four trends have been observed for RT efficiency, with some conflicting evidence amongst studies as can be seen in Tables 2, 7: (i) background tRNA has a positive impact on RT efficiency, (ii) SuperScript II is more efficient at amplifying low abundance transcripts, (iii) RT efficiency is dependent on template/gene target and (iv) RT enzyme choice contributes more to variation than technical/pipetting variation. RT enzyme plays an important role in generating both accurate and sufficient yields of cDNA, but outcomes are dependent on the target, background RNA, reagent concentrations and priming strategy. Few studies have compared priming strategy during cDNA synthesis, and even fewer have looked at the impact of primer choice on HTS output. Random hexamers tend to produce more variable yields and should be applied at high concentrations (Lekanne Deprez et al., 2002; Stangegaard et al., 2006; Werbrouck et al., 2007; Cholet et al., 2020). Gene specific primers are the most efficient in terms of yield, however they limit HTS output as they require prior knowledge of the target of interest, and do not permit a metagenomic approach (Lekanne Deprez et al., 2002; Miranda and Steward, 2017). In terms of HTS output, random hexamer priming has been shown to conserve the actual proportions of the mock community, however gene specific primers provided better coverage and Operational Taxonomic Unit (OTU) richness of the transcript in question (Schwaber et al., 2019; Cholet et al., 2020; Zucha et al., 2020). This is an important consideration in experimental design and needs to reflect the purpose of the study, i.e., to (a) to assess diversity or (b) characterize a specific target.

**Table 7:** Literature investigating the impact of RT enzyme on cDNA fidelity.

**Paper**	**RT enzymes**	**Main findings**
Waugh et al., 2015	SuperScript II and AMV RT enzymes	RNA concentration during first strand synthesis; effect of RNase H activity and PCR cycling conditions all impact both the yield and fidelity of RT
		Input RNA concentration and PCR cycles might generate a larger viral cDNA population for analysis but are likely to compromise the quality of sequencing data obtained
Yasukawa et al., 2017	HIV-1 RT, AMV, or MMLV	HIV-1 RT demonstrated lower fidelity than AMV or MMLV
Okano et al., 2018	HIV-1 RT, AMV, or MMLV	High concentrations of MgCl2 and dNTP negatively impact RT fidelity
Zucha et al., 2020	Maxima H, SuperScript II, Superscript IV, PrimeScript, SensiScript, Accuscript	Performance reproducibility was best for Maxima H and Superscript IV
Cholet et al., 2020	SuperScript II, SuperScript IV, Sesniscript, Omnicsript,	Addition of RNA mock communities into environmental RNA (before reverse transcription) can aid interpret sequencing results

#### 4.3.3. Amplicon Generation

Optimization of PCR based amplification approaches requires careful consideration of (i) the target(s) in question and (ii) the bias introduced though polymerase choice and cycling conditions. Overall trends from the relevant studies demonstrate that thermostable, high fidelity polymerases outperform the robust alternatives. AmpliTaq Gold has been commonly applied in molecular virology and yet performed poorly in all studies, regardless of target or matrix (Table 6).

#### 4.3.4. Fragmentation

Fragmentation of RNA and DNA has been observed to induce bias. The bulk of RNA-Seq studies have investigated the impact of fragmentation on relative gene expression compared to qRT-PCR measurements, rather than the detection of viral quasispecies. Bias observed is dependent on when and what type of fragmentation was applied. For fragmentation of RNA, RNase III-based fragmentation demonstrates a preference for double-stranded RNA sequences. This can result in uneven fragmentation of RNA leading to differential representation of specific regions of RNA (Adiconis et al., 2013). Parekh et al. (2016) found that a large fraction of computationally identified read duplicates were not PCR duplicates and could be explained by sampling and fragmentation bias. Fragmentation bias contributed considerably to computationally identified read duplicates and was stronger for Smart-Seq, i.e., for enzymatic fragmentation, than for TruSeq, i.e., heat fragmentation.

ClickSeq fragmenation (Routh et al., 2015; Jaworski and Routh, 2017; Wang et al., 2017) and a similar method (Wang et al., 2017) were more likely to conserve the relative abundance of the original samples due their robustness against common artifacts of HTS such as chimera formation and artefactual recombination (Routh et al., 2015). This is important as these libraries result in more accurate assessment of polymorphism frequency, species population diversity and accurate *de novo* genome assembly.

In terms of bias introduced during fragmentation of cDNA, Tn5 and other enzyme-based cDNA fragmentation methods require a precise enzyme:DNA ratio, making method optimization less straightforward than RNA fragmentation (Hrdlickova et al., 2017). When the enzymatic fragmentation is run to completion, the proportion of smaller fragments increases significantly. Furthermore, ultrasound treatment of genomic DNA could induce amplified cleavage of GC-rich areas of genome (Poptsova et al., 2014). As cDNA fragments are sequenced, the number of reads corresponding to each transcript is proportional to the number of cDNA fragments rather than the number of transcripts. Since longer transcripts are generally sheared into more fragments, more reads will be assigned to them than shorter transcripts, dismissing the possibility of relative abundance assessment of viral populations. Indeed, this fragmentation step introduces additional diversity into the starting position of the sequence (Alberti et al., 2014).

Other studies have evaluated mechanical and enzymatic fragmentation of cDNA for virus amplicon-based sequencing though with conflicting results. While Vrancken et al. (2016) determined that the fragmentation had a modest impact on sequencing results, Knierim et al. (2011) observed that while overall sequence quality was similar, enzymatic fragmentation resulted in more insertions/deletions in raw sequence reads yet outperformed mechanical fragmentation when filtering homopolymer errors.

## 5. Future Directions

Sequencing platform and bioinformatics pipelines have not been considered in this review, though it is recognized that both impact sequencing results, they are outside the scope of this review and our expertise. Most virus specific pipelines rely on k-mer frequency classification, sometimes with protein alignment based verification (Zhao et al., 2013; Roux et al., 2015; Ren et al., 2017; Alam and Chowdhury, 2020; Nayfach et al., 2020). However as Höper et al. (2020) demonstrated bioinformatic pipelines require further harmonization and standardization for diagnostic application. A comprehensive review on bioinformatic processing of viral sequencing data is required and the current pandemic (COVID-19) has placed our knowledge gaps and ability to interpret sequence data, front, and center. Our current ability to pre-empt RNA viruses of clinical concern detected from sequencing of environmental samples is limited by the need to confirm HTS results in cell culture and animal models.

**Figure 4:**
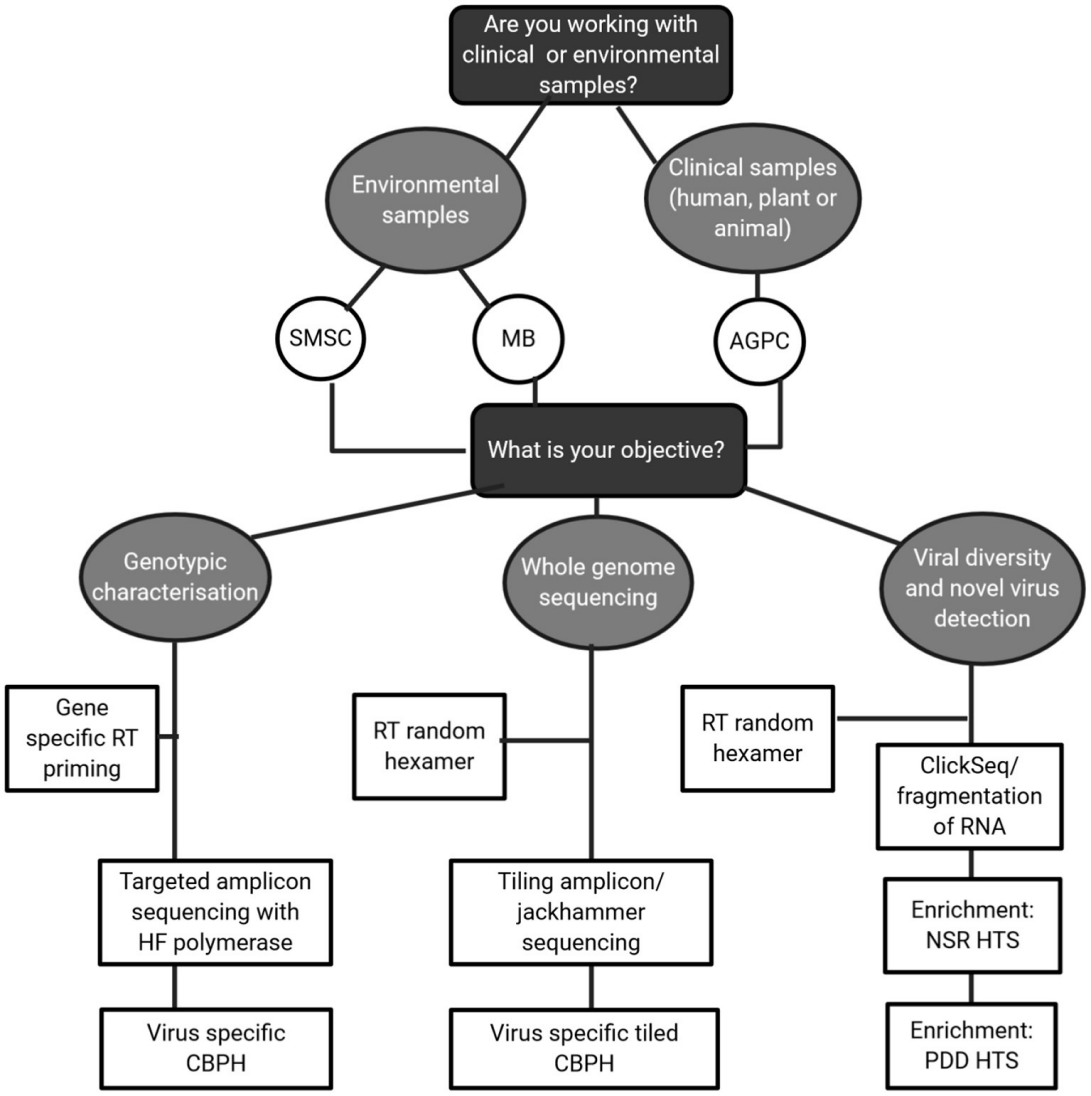
Decision tree guiding method choices for HTS of RNA viruses.

In this review, the focus has been on how to obtain high quality RNA virus sequences from complex matrices by making careful, informed choices on methodology. This is best described in the decision matrix in Figure 4. For environmental samples, likely containing low concentrations of RNA virus; method choice must be carefully balanced with the objective in mind. Clinical samples with higher viral RNA concentrations could output high quality sequences but if cheaper target amplicon sequencing answers the research questions in mind then it is not necessary. For method steps such as RT and fragmentation, a target specific approach should be taken and current literature surveyed of indications on performance, particularly for RT priming approaches. While agnostic approaches are theoretically preferable, they may not provide sufficient coverage of viral genomes for classification, thus limiting their usefulness as a standard sequencing approach. Therefore, intermediaries such as capture probe hybridization and tiling/jackhammer amplicon approaches should be strongly considered as initial approaches and complemented with long read sequencing.

Without controls, results are meaningless. The inclusion of whole process controls, internal process controls such as spike-in DNA and negative controls provide greater certainty on the obtained sequencing reads, particularly in the case of shotgun metagenomics. Novel RNA viruses or variants should be confirmed by PCR and/or Sanger sequencing and relative abundance should be not relied upon as a quantitative measure.

While it is challenging to obtain high quality sequences from environmental samples, the information that could be gleaned is essential for maintaining public health. From developing new PCR/qPCR assays based on recent sequencing data, to monitoring antigenic drift and recombination, identifying new transmission pathways, hosts and viruses or pre-empting RNA viruses and variants of clinical concern in a One Health paradigm, the list of potential benefits goes on. HTS has much to offer to the field of environmental virology but in incorporating it into the arsenal of molecular tools already utilized, it is important to be aware of the challenges and biases and to circumvent these by considering both the matrix and target virus(es) in question.

## Author Contributions

AF reviewed the literature and compiled the review. AR revised the initial drafts. SK, HO'S, PC, and FC reviewed the final draft. All authors contributed to the article and approved the submitted version.

## Conflict of Interest

The authors declare that the research was conducted in the absence of any commercial or financial relationships that could be construed as a potential conflict of interest.

## Abbreviations

AGPC: Acid guanidinium thiocyanate phenol chloroform
CBPH: Capture based probe hybridization
cDNA: complementary cDNA
DMSO: Dimethyl sulphoxide
DNA: deoxyribonucleic acid
ERCC: External RNA Control Consortium
HTS: High Throughput Sequencing
ITS: Internal transcribed spacer
LNA: Locked nucleic acid
MB: Magnetic beads
MIQE: Minimum Information for Publication of Quantiative Real-Time PCR Experiments
mRNA: messenger RNA
NSR: Not so random
OUT: Operational Taxonomic Unit
PDD: Probe directed degradation
PEG: Polyethylene glycol
qRT-PCR: quantitative Reverse Transcription Polymerase Chain Reaction
RCA: Rolling Circle Amplification
RIN: RNA Integrity number
RNA: ribonucleic acid
RT-PCR: Reverse Transcription Polymerase Chain Reaction
SIRVs: Spike in RNA variants
SISPA: Sequence Independent, Single Primer Amplification
SMSC: Silica membrane based spin column technology
SNV: Single Nucleotide Variants
SPIA: Single Primer Isothermal Amplificaiton
ssRNA: single stranded RNA.
